# Molecular profiling of lung adenosquamous carcinoma: hybrid or genuine type?

**DOI:** 10.18632/oncotarget.4163

**Published:** 2015-06-03

**Authors:** Erik Vassella, Stephanie Langsch, Matthias S. Dettmer, Cornelia Schlup, Maja Neuenschwander, Milo Frattini, Mathias Gugger, Stephan C. Schäfer

**Affiliations:** ^1^ Institute of Pathology, University of Bern, Bern, Switzerland; ^2^ Institute of Pathology, Locarno, Switzerland; ^3^ Promed SA Laboratoire Medical, Fribourg, Switzerland; ^4^ Institute of Pathology, University Hospital of Cologne, Cologne, Germany

**Keywords:** adenosquamous carcinoma, lung, next-generation sequencing, therapy-relevant mutation, microRNA

## Abstract

Lung adenosquamous carcinoma is a particular subtype of non-small cell lung carcinoma that is defined by the coexistence of adenocarcinoma and squamous cell carcinoma components. The aim of this study was to assess the mutational profile in each component of 16 adenosquamous carcinoma samples from a Caucasian population by a combination of next generation sequencing using the cancer hotspot panel as well as the colon and lung cancer panel and FISH. Identified mutations were confirmed by Sanger sequencing of DNA from cancer cells of each component collected by Laser Capture microdissection. Mutations typical for adenocarcinoma as well as squamous cell carcinoma were identified. Driver mutations were predominantly in the trunk suggesting a monoclonal origin of adenosquamous carcinoma. Most remarkably, *EGFR* mutations and mutations in the PI3K signaling pathway, which accounted for 30% and 25% of tumors respectively, were more prevalent while *KRAS* mutations were less prevalent than expected for a Caucasian population. Surprisingly, expression of classifier miR-205 was intermediate between that of classical adenocarcinoma and squamous cell carcinoma suggesting that adenosquamous carcinoma is a transitional stage between these tumor types. The high prevalence of therapy-relevant targets opens new options of therapeutic intervention for adenosquamous carcinoma patients.

## INTRODUCTION

Lung cancer is the leading cause of cancer related death worldwide with a mean 5 year survival rate of less than 15% [1]. 85% of lung cancer is classified as non-small cell lung carcinoma (NSCLC). The two predominant histological subtypes are adenocarcinoma (AD), which account for 50% of NSCLC, and squamous cell carcinoma (SQ), which account for 40% of NSCLC [2]. AD have glandular histology and intracellular mucous production and express thyroid transcription factor 1 (TTF1) consistent with an origin in the distal lung. By contrast, SQ, which arise in more proximal airways, are more reminiscent of pseudostratified columnar epithelium of the upper airways and are characterized by keratinisation, intercellular desmosome formation and expression of p63 [3] and p40 [4]. In addition, both entities are characterized by a different set of driver mutations. Mutations of the oncogenes *KRAS* (25–40%), *EGFR* (10–15%), *BRAF* (2–4%), and *HER2* (2%), and translocations of *ALK* (5–7%) and *ROS1* (2%) are found predominantly in AD at frequencies indicated in brackets, most of which are targets for currently available or potential targeted therapies [5]. Potentially targetable driver mutations are less frequent in SQ than in AD. These include mutations of the discoid-containing receptor 2 (*DDR2*) (3.8%), *PTEN* (10%) and *PIK3CA* (16%), or amplification of *FGFR1* (20%) [6, 7].

Adenosquamous carcinoma (ADSQ) of the lung is a rare subtype of NSCLC that accounts for 2–4% of lung cancer [8]. This type is characterized by the morphological presence of both SQ and AD patterns, each comprising at least 10% of the total tumor volume. The two components represent the typical immunoprofile of AD and SQ differentiation in the lung, i.e. TTF1 and napsin positivity (80% and 58% respectively) for the AD component and p63 and CK5/6 positivity (100% and 73% respectively) for the SQ component [9]. p40 is considered superior to p63 in terms of specificity of classical SQ [4], but whether this marker allows for the discrimination of AD and SQ components of ADSQ is not well studied. The prognosis of ADSQ is generally worse compared to classical AD or SQ, independent of the ethnic background [10–12].

ADSQ is a striking example of a morphologically dichotomous tumor whose genomic landscape has yet to be systematically probed for its contributory role to this dichotomy. In classical lung AD, multiregional sequencing revealed that the majority of mutations of cancer genes were trunk mutations, which represent ubiquitous mutations present in all regions of the tumor, and one out of 21 mutations within cancer genes was a branch mutation [13]. The latter class of mutations represents heterogeneous mutations present only in one region of the tumor. Intratumoral heterogeneity may have an impact on biopsy strategy, as single biopsies may be inadequate for identifying all cancer gene mutations. These findings may have consequences on treatment planning since important drug targets may be missed. The degree of tumor heterogeneity correlates with the likelihood of postsurgical relapse in patients with localized lung cancer [13].

The aim of this study was to assess tumor heterogeneity of ADSQ by a combination of next generation sequencing (NGS) using the Cancer hotspot panel as well as the Colon and Lung Cancer panel and fluorescence in situ (FISH) analysis. To verify mutations identified by NGS, tumor cells were collected from AD and SQ components by laser capture microdissection and analyzed by Sanger sequencing. This allowed us to assess if each component harbors a different set of mutations. To our knowledge, this is the first comprehensive study assessing the mutational status of cancer-related genes in ADSQ.

## RESULTS

### Patient's collective

A total of 16 patients diagnosed with adenosquamous carcinoma of the lung at the Institutes of Pathology in Bern and Locarno were enrolled in this analysis. The characteristics of the patients are shown in Supplementary Table 1. The mean age was 76 years and most patients were male. Thirty percent of tumors showed evidence of lymph node metastasis and the majority of tumors showed moderately differentiated histology.

### Analysis of AD and SQ components of ADSQ

**Figure 1:**
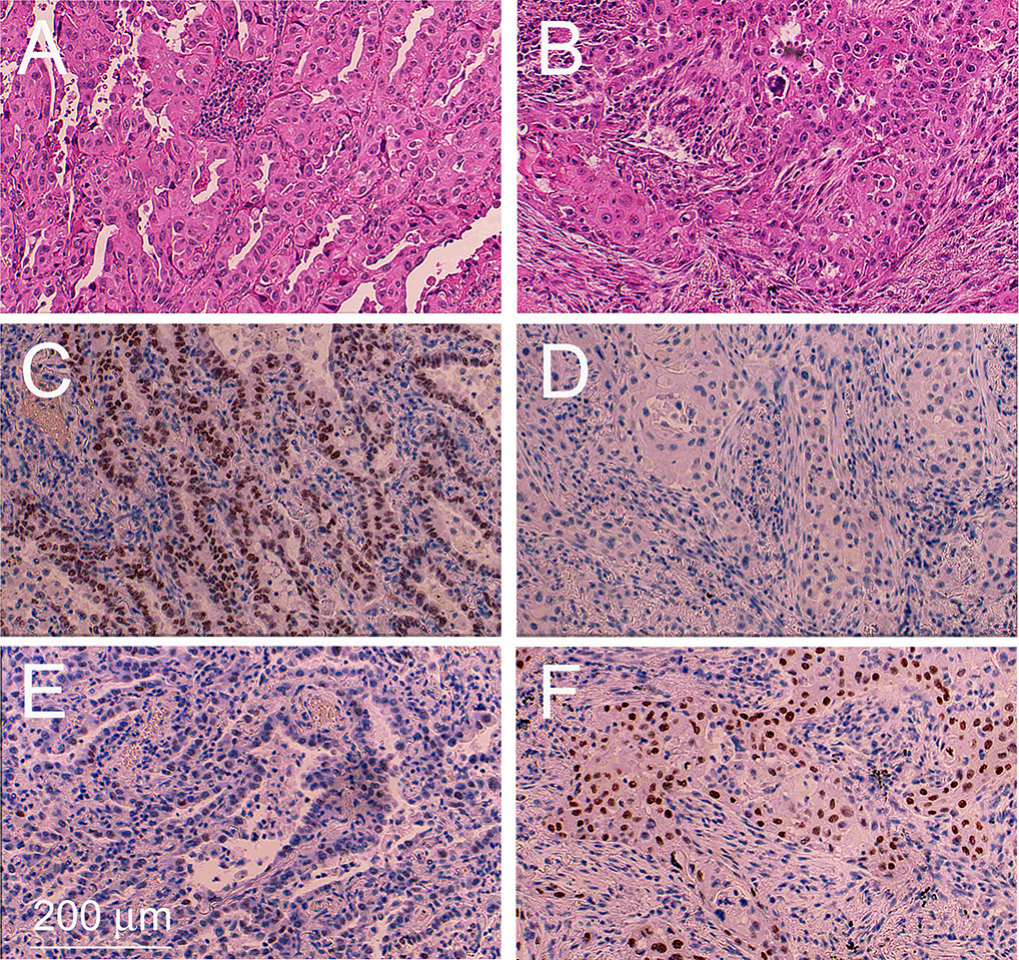
Example of an adenosquamous carcinoma tissue sample Tissue sections encompassing the adenocarcinoma component **A, C, E.** and squamous cell component **B, D, F.** were stained with H&E (A, B), immunostained with a monoclonal antibody against TTF1 (C, D) and immunostained with a monoclonal antibody against p63 (E, F).

Serial tissue sections from paraffin blocks were performed as outlined in Supplementary Figure 1. The tumor region encompassing the AD and SQ components were defined by histological and immunohistochemical criteria. Morphological criteria for the AD component were glandular histology and intracellular mucous production. Morphological criteria for the SQ component were pseudostratified columnar histology, keratinisation and formation of intercellular desmosomes (Figure 1A and 1B) (see WHO guide lines [1–3]).

Immunohistochemical staining for Thyroid transcription factor 1 (TTF1), a marker specific for AD of the lung [9], gave rise to a significantly stronger signal in the AD component compared to the SQ component in 13 out of 16 ADSQ (Table 1 and Figure 1C and 1D). Tumors #3, 9 and 10 revealed either no staining or equal staining for TTF1 in both components. These tumors were analyzed for the expression of napsin, an alternative marker for classical AD. All tumors showed high level expression of napsin in the AD component, but no expression in the SQ component (Table 1). By contrast, all ADSQ samples revealed much stronger staining for p63 in the SQ component than in the AD component (Table 1 and Figure 1E and 1F). Consistent with this result, the p40 marker, which is considered a more specific marker than p63, gave rise to intensive staining of the SQ component but no staining of the AD component in 15/16 ADSQ (Table 1). In conclusion, all tumors could be clearly classified as ADSQ based on immunohistochemical stainings.

**Table 1:** Characterization of adenosquamous carcinomas by immunohistochemistry and FISH

Tumor	Morphological classification	Immunohistochemistry					FISH		
		TTF-1	p63	napsin	p40	TP53	*ALK*	*ROS1*	*EGFR*
1	AD	++^1^	–	na	–	+	–	–	–
	SQ	+	++	na	++	+	–	–	–
2	AD	++	–	na	–	–	–	–	++
	SQ	–	+	na	++	–	–	–	–
3	AD	+	–	++	–	++	–	–	–
	SQ	+	++	–	++	++	–	–	–
4	AD	+	–	na	–	++	–	–	–
	SQ	–	++	na	++	++	–	–	–
5	AD	++	+	na	–	+	–	–	–
	SQ	–	++	na	+	+	–	–	–
6	AD	+	–	na	–	+	–	–	–
	SQ	–	++	na	++	+	–	–	–
7	AD	++	+	na	–	+	–	–	–
	SQ	–	++	na	++	+	–	–	–
8	AD	+	+	na	+	++	–	–	–
	SQ	–	++	na	++	++	–	–	–
9	AD	–	–	++	–	–	–	–	–
	SQ	–	++	–	++	–	–	–	–
10	AD	–	–	++	–	+	–	–	–
	SQ	–	++	–	++	+	–	–	–
11	AD	++	–	na	–	+	–	–	–
	SQ	–	++	na	++	+	–	–	–
12	AD	+	–	na	–	+	–	–	–
	SQ	–	++	na	++	+	–	–	–
13	AD	+	–	na	–	–	–	–	–
	SQ	–	++	na	++	++	–	–	–
14	AD	+	–	na	–	–	–	–	–
	SQ	–	++	na	++	–	–	–	–
15	AD	+	–	na	–	–	–	–	–
	SQ	–	++	na	–	–	–	–	–
16	AD	+	–	na	–	–	–	–	–
	SQ	–	++	na	++	++	–	–	–

1 –, negative; +, weak positive; ++, strong positive or amplified; na, not analyzed

### Mutational analysis of AD and SQ components of ADSQ

In a next step, the mutational profile of each component of ADSQ was assessed. To this end, regions encompassing AD and SQ components, as indicated by immunohistochemical stainings, were macroscopically dissected from unstained tissue slides. Regions containing > 50% normal lung tissue or inflammatory infiltrates were excluded from the analysis. DNA was extracted from corresponding regions and subjected to NGS using the Cancer Hotspot panel, which covers most of the important cancer genes. DNA was also subjected to NGS using the Colon and Lung Cancer Research panel, which allows the assessment of the mutational status of additional genes including *DDR2*, *MAP2K1* and *FBX7*, which are not covered by the Cancer Hotspot panel. Sequence analysis revealed an average number of 480, 000 mapped reads and an average mean depth of 2, 000 reads. Variant calling was filtered for nonsynonymous SNPs, indels and spliced variants. FFPE material may result in false positive low frequency (< 5%) calls owing to fixation artifacts. To remove false positive calls, variant allele frequency threshold was set to 5%. Common germ-line mutations that are predicted to have no effect on the protein function, as indicated by SIFT/polyphen analysis, were also discarded.

Supplementary Table 2 shows all predicted nonsynonymous somatic changes that were identified by NGS using the Cancer Hotspot and Colon and Lung Cancer panels. A total of 26 somatic mutations were identified in 15 out of 16 tumors while one tumor revealed no mutation. Among the identified mutations, 3 indel mutations, 2 splice-site mutations and 21 nonsynonymous SNPs were obtained. Nine out of 26 mutations revealed variant read frequencies that were > 3 times higher in one component compared to the other component of the same tumor sample, suggesting that these mutations are branch mutations (Supplementary Table 2). These include *TP53* p.T155I, p.Y126C and p.P190L mutations in samples #4 and #13, *CDKN2A* p.W110* mutation in #5, *PIK3CA* p.E545K mutation in #4 and 10, and *RB1* p.G203fs*8 and PTEN p.Q171* mutations in #16. All other mutations seem to be trunk mutations as they gave rise to variant read frequencies that were similar in both components.

Although corresponding regions were carefully macrodissected, it cannot be excluded that some DNA samples may be cross-contaminated by the DNA from the other component. To assess this possibility, cells were collected by laser capture microdissection from each component and analyzed by Sanger sequencing for mutations identified by NGS. To assess if identified mutations were indeed of somatic origin, normal tissue collected from the same patient was also included. There was a high agreement between the results obtained by NGS and Sanger sequencing: not only were the same mutations identified but also the assignment of these mutations to the trunk or branches, respectively, was consistent between both sequencing methods (Supplementary Table 2).

**Figure 2:**
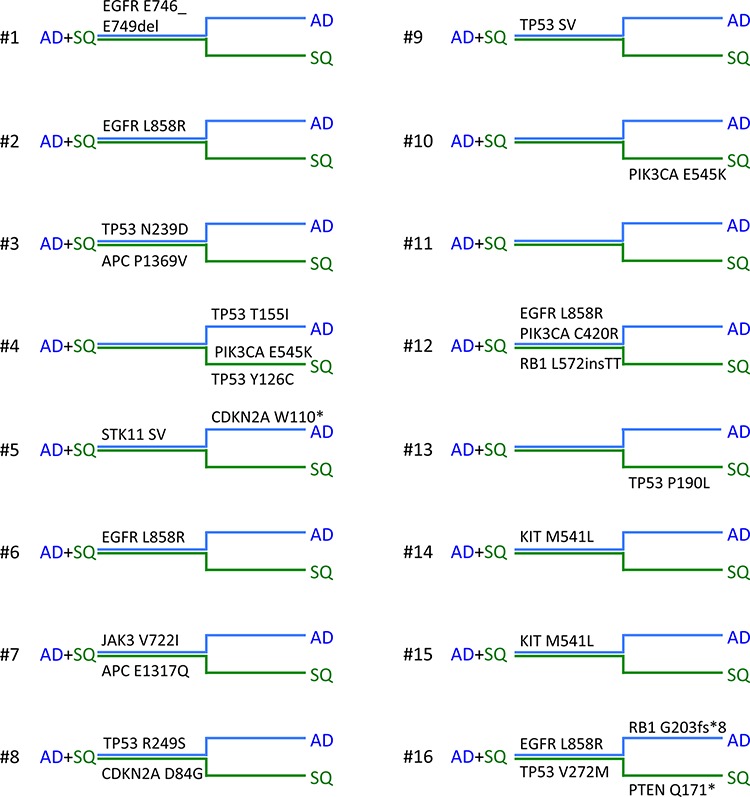
Mutational landscape of adenosquamous carcinomas Horizontal phylogenetic trees are shown for all ADSQ analyzed in this study. Mutations common to both tumor components are represented by the trunk (AD + SQ, close parallel lines) and mutations only found in the AD (blue line) or SQ (green line) component are represented by branches. Individual ADSQ tumors are indicated by numbers.

**Figure 3:**
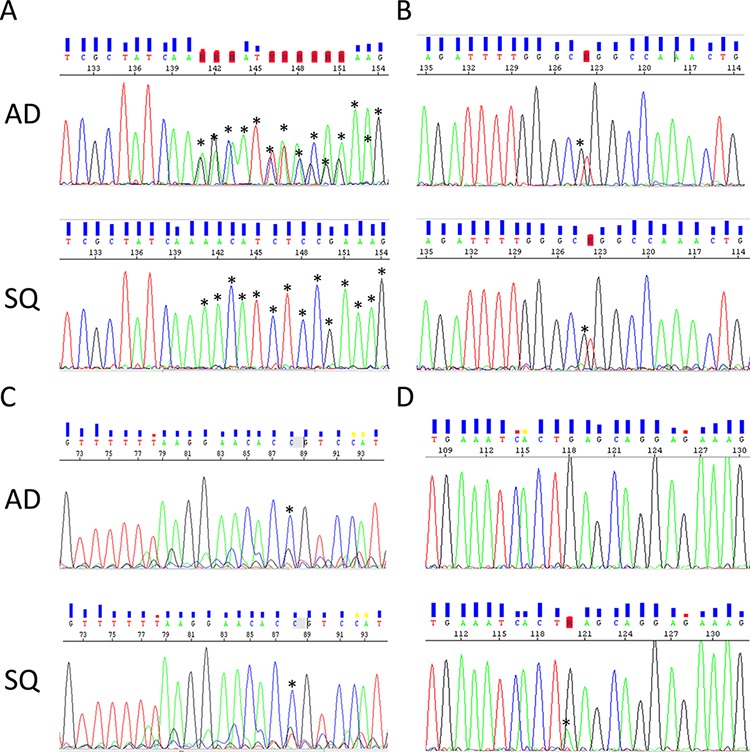
Representative electropherograms of *EGFR* and *PIK3CA* genes from corresponding AD (upper panel) and SQ (lower panel) components **A.** *EGFR* p.E746_E749del deletion in tumor #1. **B.** *EGFR* p.L858R mutation in tumor #12. **C.** *PIK3CA* p.C420R mutation in tumor #12. **D.** *PIK3CA* E545K mutation in tumor #10. Mutations in (A–C) are common to both components and the mutation in (D) is unique to the SQ component. Asterisks indicate alterations to the wild-type sequence.

Somatic mutations identified in this study are presented in Figure 2. Our results indicate that mutations in the *EGFR* signaling pathway are among the most prominent mutations in ADSQ. Five out of 16 tumors harbored bona fide *EGFR* mutations including a p.E746_E750 deletion in sample #1 and a p.L858R missense mutation in samples # 2, 6, 12 and 16 (Supplementary Table 2, Figure 2 and Figure 3A, 3B). These mutations confer increased sensitivity to tyrosine kinase inhibitors [14, 15]. In each case, the same *EGFR* mutation was detected in both components of the tumor (Figure 2 and Figure 3A, 3B) indicating a monoclonal origin of ADSQ. Interestingly, *EGFR* mutations were more prevalent than expected for a Caucasian population. By contrast, *KRAS* mutations, which are present in 25–40% of AD [5], were not detected in our collective of ADSQ samples.

Mutations in the *PI3K* signaling pathway are typical for SQ [7]. Four ADSQ tumors harbored mutations in the *PIK3* pathway; one tumor harbored a p.C420R mutation in the C2 region and two tumors harbored a p.E545K mutation in the helical region of the *PIK3CA* gene, and one tumor harbored a p.Q171* mutation in the *PTEN* gene. All three mutations are predicted to result in constitutive activity of the PI3K signaling pathway [16]. In tumor sample #12, the same mutation was detected in both components (Figure 2 and Figure 3C) while tumor samples #4, #10 and #16 harbored mutations that were only present in one, but not the other component (Figure 2 and Figure 3D). Thus ADSQ show mutations typical for both classical AD and SQ.

**Figure 4:**
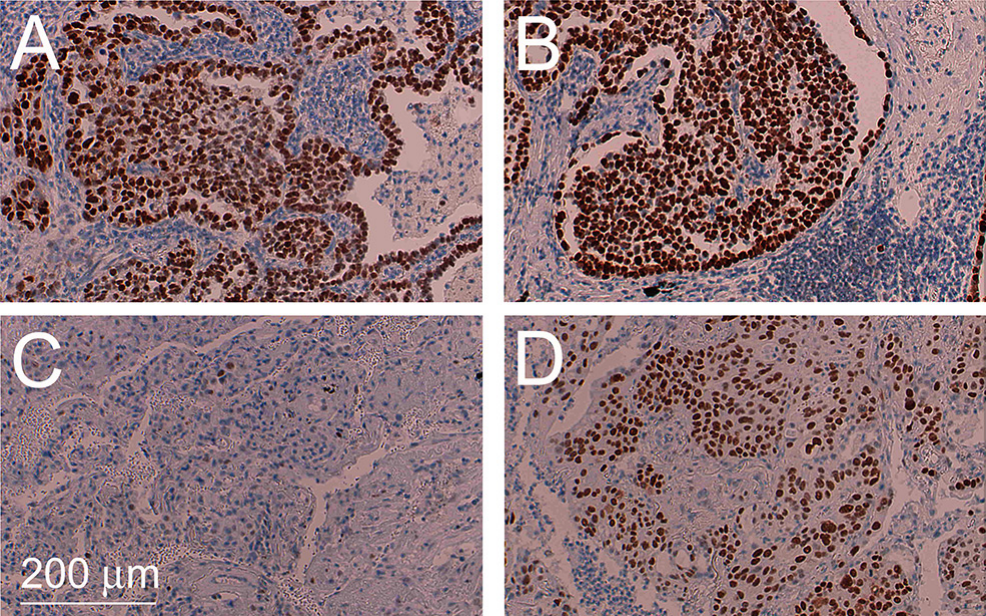
Intratumoral heterogeneity of TP53 mutations Immunohistochemical staining for TP53 of AD component of tumor #4 harboring a *TP53* p.T155I mutation **A.** corresponding SQ component harboring a *TP53* p.Y126C mutation **B.** AD component of tumor #13 containing the wild-type sequence **C.** and corresponding SQ component harboring a *TP53* p.P190L mutation **D.**

*TP53* mutations were also common in our collective of ADSQ: 6 out of 16 tumors harbored *TP53* mutations, 4 of which were trunk mutations while 2 were branch mutations. In particular, both components of tumor #4 contained different *TP53* mutations. This suggests that *TP53* mutations can either occur before or after separation of the AD and SQ components from a common precursor. Most hotspot mutations of the *TP53* gene including some of the mutations identified in this study are gain of function mutations, which lead to increased expression of p53 protein in the tumor tissue [17]. To assess if the expression pattern for p53 reflects the mutational profile, immunohistochemistry was performed using a monoclonal antibody directed against p53. As shown in Table 1 and Figure 4A–4D, tumor samples #3, 4, 8, 13 and 16, which contained gain of function mutations of the *TP53* genes, also showed enhanced expression of the p53 protein. Most strikingly, tumors #3, 4, 8 and 16 which either contained a *TP53* mutation in the trunk or independent *TP53* mutations in the branches, showed enhanced p53 expression in both components while tumor #13, which contained a *TP53* mutation only in the SQ component, showed enhanced expression exclusively in this component. In contrast, tumor #9, which contained a loss of function mutation of the *TP53* gene owing to a A > T transversion at the canonical 5′ splice site of exon 6 in both components, gave rise to no p53 protein expression (Table 1).

In addition, mutations in cell cycle genes including those in *Rb* and *CDKN2A* were identified in 3 tumor samples (#5, 8 and 16). These mutations are predicted to lead to enhanced cell cycle progression of tumor cells. It remains to be shown if the p.P1369V missense mutation in the *APC* gene in tumor #3 results in enhanced activity of the β-catenin pathway. A G > C transversion at the 5′ splice site of exon 4 of *STK11* was also identified in both components of tumor #5. Finally, a p.M541L mutation in the transmembrane domain of the *KIT* gene and a p.V722I mutation in the *JAK3* gene were identified (samples #7, 14 and 15, Figure 2). These sequence alterations have been identified as somatic mutations as well as sequence variants occurring in the normal population, and it is not clear whether or not these sequence alterations may affect protein functions. The mutations identified in our collective were clearly somatic mutations as they were not present in normal tissue collected from the same patient.

*ALK* and *ROS1* translocations are also important oncogenic drivers of NSCLC [5]. Since these genomic alterations cannot be detected by NGS using the Cancer hotspot or Colon and Lung Cancer panels, FISH analysis was performed. However, no *ALK* or *ROS1* translocation was detected in our collective of ADSQ (Table 1). Tumor #2 gave rise to *EGFR* amplification in the AD component, but not the SQ component.

### Analysis of classifier miR-205 in ADSQ tumor samples

**Figure 5:**
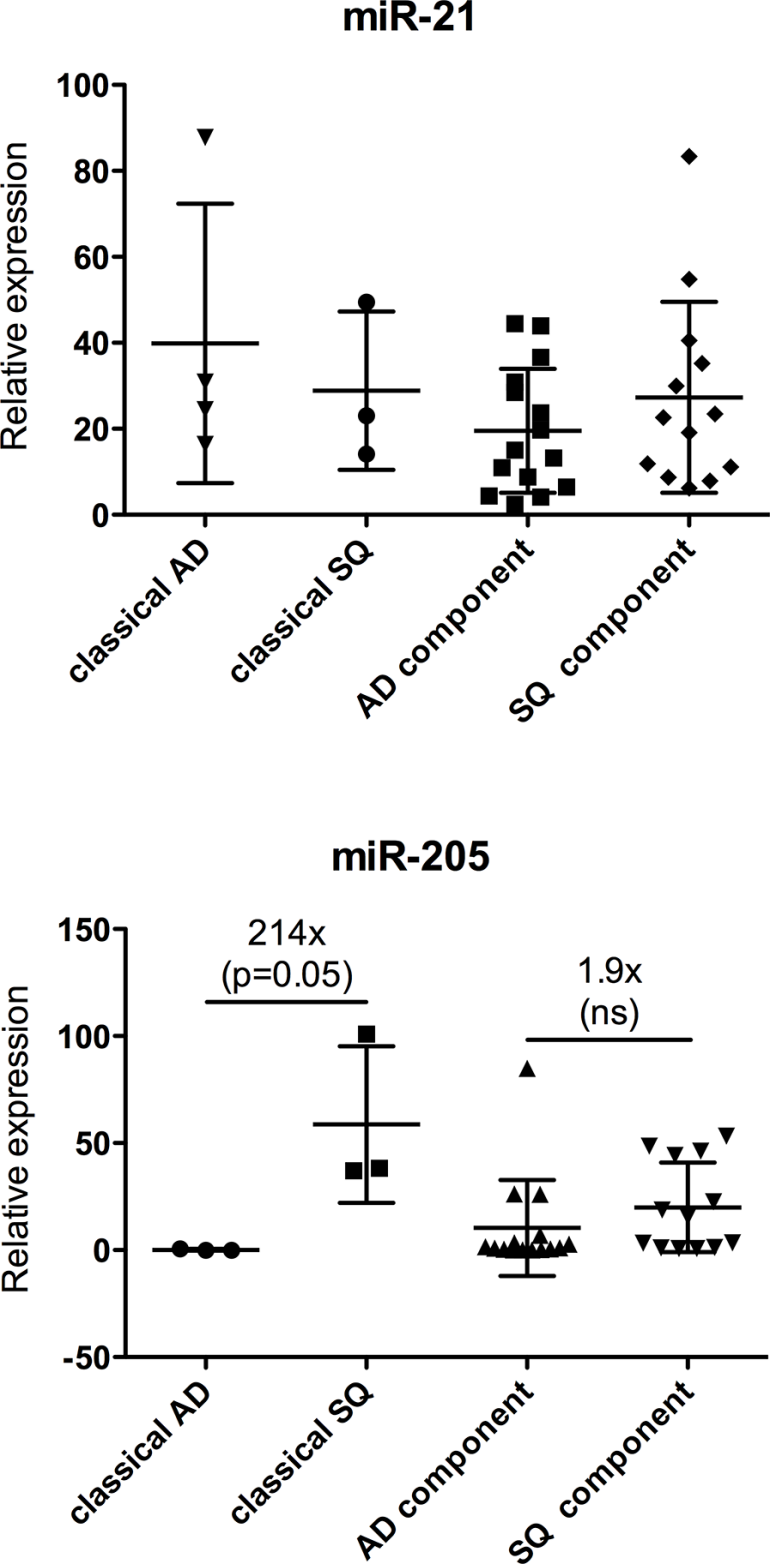
Expression analysis of miR-21 (upper panel) and miR-205 (lower panel) in ADSQ components Expression of miR-21 or miR-205 relative to the expression of RNU-48 is shown for classical AD and SQ and for AD and SQ components of ADSQ. The difference in the expression of miR-205 between classical AD and SQ was statistically significant (*p* = 0.05).

MicroRNAs are important regulators of differentiation and their expression profile correlates significantly with the state of differentiation of various tissues [18]. Hence miRNAs proved to be very useful for tumor classification [19]. To assess if ADSQ components reflect the expression pattern of classical AD and SQ, classifier miR-205, which is upregulated in SQ [20], and miR-21, which is upregulated in both tumor subtypes, were analyzed. As shown in Figure 5, miR-21 was expressed at similar levels in both components of ADSQ as well as in classical AD and SQ. In agreement with published results, the expression level of miR-205 was 200 times higher in classical SQ than in classical AD (*p* = 0.05). In contrast, no significant difference in the expression level was observed between AD and SQ components of ADSQ.

In conclusion, although AD and SQ components clearly differ by morphological as well as immunohistochemical criteria, they cannot be discriminated by means of mutational profiling of cancer genes or by expression analysis using classifier miRNAs.

## DISCUSSION

Only a few studies exist which assessed the mutational profile of ADSQ [21–25]. In almost all studies Asian populations were analyzed, but it is not known if this ethnic group may exhibit a different mutational profile compared to Caucasian populations. In most cases mutational analysis was restricted to *EGFR* or *KRAS*. We are the first to provide a mutational analysis of ADSQ from Caucasian patients using an extended set of cancer-related genes including those which are frequently mutated in classical AD or SQ. All somatic mutations which were identified by NGS were confirmed by Sanger sequencing in cancer cells collected from each component by laser capture microdissection. This approach ensures that there is little cross-contamination between ADSQ components and therefore allows an unequivocal assignment of mutations to either component. In contrast, most other studies used macroscopically dissected material, which may be potentially cross-contaminated.

We show for the first time that ADSQ harbor driver mutations, which are either specific for classical AD or SQ including mutations in *EGFR* or genes in the *PI3K* pathway. The surprisingly high prevalence of EGFR mutations in ADSQ is a key finding of this study. This has significant therapeutic implications: small tyrosine kinase inhibitors gefitinib and erlotinib are clinically effective for the treatment of NSCLC harboring activating EGFR mutations [14, 15]. Thus, although ADSQ is more aggressive compared to classical AD or SQ [12], more patients diagnosed with ADSQ may benefit from targeted therapy. In contrast, *KRAS* mutations were less prevalent than expected for a Caucasian population. The finding that EGFR was more prevalent and KRAS was less prevalent in ADSQ than in classical AD of patients of the Caucasian ethnic group suggests that carcinogenesis of ADSQ may be more similar between Western and Asian populations compared to classical AD. Tochigi et al. [23] reported similar *EGFR* mutation frequencies in classical AD and ADSQ within a Caucasian ethnic group which is in contrast to our findings. The reason for the discrepancy of these results is not clear, but it may either be explained by the relatively small cohort of patients used in these studies or, alternatively, by the analysis of different cohorts with specific risk factors. We show that *EGFR* mutations are present in both components suggesting that patients with ADSQ may be accessible to therapy using *EGFR* tyrosine kinase inhibitors. Indeed, several case studies reported good therapy responses of patients with ADSQ to *EGFR* inhibitors [24].

In addition to *EGFR* mutations, we observed a high incidence of mutations in genes of the *PI3K* signaling pathway, some of which were trunk mutations and some were branch mutations occurring in the AD or SQ component. Preclinical studies suggested that patients with *PIK3CA* or *PTEN* mutations may benefit from pan-class I PI3K inhibitors [26, 27]. In addition, lung cancer cell lines harboring *PIK3CA* or *PTEN* mutations were sensitive to dual *PIK3CA*/*mTOR* inhibitors [28, 29], which raises the hope that patients with mutations in the PI3K pathway may benefit from this type of targeted therapy in the future. Introduction of activating *PIK3CA* mutants into *EGFR* mutant cell lines conferred resistance to *EGFR* inhibitors [29]. Based on these results we may conclude that patient #12, who contains an *EGFR* L858R mutation as well as a *PIK3CA* C420R mutation in the tumor DNA, may be less responsive to *EGFR* or *PIK3CA* inhibitors, but this has not yet been confirmed by clinical studies. It is possible, however, that this patient may benefit from new combinations of targeted drugs.

Our results indicate that driver mutations were normally in the trunk. This suggests that ADSQ are of monoclonal origin, which is in agreement with findings obtained by others [22, 23, 25, 30–32] and which may also be true for other heterogeneous lung tumors [31]. The molecular results support the histopathologist historical “morphology only” view of ADSQ being a real entity fitting in the “lung heterogeneity concept” [33, 34]. Mather et al. [35] reported the isolation of cancer stem like cells from human ADSQ that recapitulated all features of ADSQ in mouse xenograft experiments, consistent with a monoclonal origin of ADSQ. However, the molecular mechanism of (trans)-differentiation into AD or SQ components is not clear. Loss of *STK11* or targeted deletion of *PTEN* or *TGFβR1* in the oncogenic *KRAS^G12D^* mouse model gave rise to ADSQ-like tumors [36]. Emerging tumors initially showed AD histology and expressed AD-specific markers, but *STK11*-deficient lung AD progressively transdifferentiated to SQ [37], suggesting that ADSQ are a transitional state between AD to SQ differentiation. Conversely, expression of SQ-related antigen in the AD component of ADSQ was higher while Mucin1 was lower than in classical AD suggesting a transition from SQ to AD [38]. Our findings that ADSQ can harbor mutations typically found in AD or SQ, respectively, may suggest that ADSQ have developed either from an AD-like or SQ-like precursor cell. Five ADSQ tumors (#1, 2, 6, 12 and 16) harbored EGFR mutations suggesting a transition from AD to SQ. In contrast, tumors #4, 10 and 16 harbored a PIK3CA/PTEN mutation in the SQ branch suggesting that acquisition of this mutation induced the transition into the SQ component. Expression of classifier miR-205 in the AD and SQ components of ADSQ was intermediate between that of classical AD and SQ consistent with the finding that ADSQ is a transitional state between classical AD and SQ, but this possibility has to be confirmed by extended miRNA expression profiling or methylation analysis. By contrast, both components of ADSQ also clearly show hallmarks of classical AD or SQ as indicated by histomorphological criteria as well as expression of TTF1, napsin, p63 or p40.

Multiregional sequencing of classical AD revealed that only 1 out of 21 cancer genes were in the branch, while all other mutations were trunk mutations [13]. In contrast, our study revealed that one out of four mutations was a branch mutation. In particular, *TP53* mutations were often identified as branch mutations. These results indicate that separation of AD and SQ components may have occurred very early during the development of ADSQ. The fact that ADSQ reveals a high incidence of branch mutations, makes it an attractive model for studying tumor heterogeneity.

In conclusion, we show that ADSQ harbors driver mutations specific for both classical AD and SQ. Owing to the high prevalence of these mutations, a higher proportion of patients may benefit form targeted therapy than expected for a Caucasian population. In addition, our results have diagnostic implications, especially in advanced tumors which are not resected and where only small biopsies are available for molecular testing: since the most relevant targetable driver mutations are mainly found in the trunk, they may not be missed by mutational analysis in such specimens. However, a problem that arises using small biopsies is that ADSQ can present as SQ if the AD component is missed. Consequently molecular testing for the most important drugable targets in AD like *EGFR, ALK* or *ROS1* will not be performed, leaving the patient without this therapeutic option. Nevertheless, this issue may become less relevant in the future, when all NSCLC patients are analyzed by NGS.

## MATERIALS AND METHODS

### Patients and samples

The ethical boards of the Inselspital Bern and the institute of Pathology of Locarno have approved the study as part of the general approval for research on formalin fixed paraffin embedded tissue (KEK Nr. 200/2014). Using the full text research tool of our database software (Pathowin +), we selected 35 cases of adenosquamous carcinoma patients diagnosed in 1993–2007. All cases were re-reviewed by two experienced pathologists for confirmation of tumor histology following the guidelines of the WHO 2004 classification [2]. After review of the histological slides 16 cases of operation specimens were selected for the study. Nineteen cases were excluded due to pretreatment, extensive necrosis or ambiguous immunohistochemical staining.

### Serial tissue sectioning, laser capture microdissection and nucleic acid extraction

Serial 3–6 μm tissue sections were performed from paraffin blocks as outlined in Supplementary Figure 1. AD and SQ components of each tumor were macroscopically dissected for DNA and RNA extraction using the QIAamp DNA Micro Kit (QIAGEN) and RecoverALL Total Nucleic Acid Isolation kit (Ambion), respectively, following the manufacturer's instructions. Methylol groups that are covalently bound to nucleic acids during formalin treatment were cleaved from nucleic acids as described [39].

Laser capture microdissection was performed on 6 μm sections on Polyethylene Naphthalate (PEN)-membrane slides [39]. Sections were deparaffinized and stained with cresyl violet following the manufacturer's instruction (Zeiss). Approximately 1000 tumor cells for each component were captured onto an adhesive cap using a PALM Microbeam (PALM, Zeiss). DNA was extracted from the dissected material as described above.

### Immunohistochemistry

Three μm formalin-fixed, paraffin-embedded sections were immunostained for TTF1 (Abcam, clone 2Cla, 1:50, pretreated with citrate buffer for 20 min at 100°C), p63 (Leica-Novocastra, clone 7 Jul, 1:40, pretreated with bond 2 for 30 min at 100°C), napsin (Leica-Novocastra, clone IP64, 1:400, pretreated with bond 2 for 30 min at 95°C), p40 (Biocare Medical, polyclonal antibody, 1:100) and TP53 (Dako, clone DO-7, 1:200, pretreated with bond 2 for 20 min at 95°C) using a Bond Max autostainer (LEICA Bond III platform) from Leica Microsystems (Wetzlar, Germany) and counterstained with haematoxylin. Mouse IgG1 (Dako, 1:20) was used as a negative control.

### Fluorescence *in-situ* hybridization

The gene status of *ALK* (Vysis LSI ALK Dual Color, break apart rearrangement probe, Abbott Molecular, Des Plaines, IL, USA), *ROS1* (ZytoLight SPEC ROS1 Dual Color Break Apart Probe, Zytovision, Bremerhaven, Germany) and *EGFR* (Vysis LSI EGFR spectrumOrange/CEP7 spectrumGreen Probe, Abbott Molecular) were evaluated using commercially available FISH probes. 2–3 μm tissue sections were deparaffinized and pretreated using the commercial pretreatment kit Vysis (Abbott Molecular) and hybridized with the probe overnight in a humidified chamber at 37°C. Post hybridisation washes were performed using the Pathvysion kit (Abbott Molecular) and counterstained with DAPI. Thresholds for a positive signal constellation for the *ALK* or *ROS1* probes were set to 15%. Threshold for a positive *EGFR* signal, as indicated by an *EGFR* to CEP7 ratio > 2.0, was set to 10%. At least 60 cells were counted.

### Library preparation and semiconductor sequencing

For library preparation, multiplex PCR was performed using the Ion Ampliseq Cancer Hotspot Panel v2 (Life Technologies, Zug). This panel consists of a single pool of 207 primer pairs and results in the amplification of the following genes: *ABL1, EZH2, JAK3, PTEN, AKT1, FBXW7, IDH2, PTPN11, ALK, FGFR1, KDR, RB1, APC, FGFR2, KIT, RET, ATM, FGFR3, KRAS, SMAD4, BRAF, FLT3, MET, SMARCB1, CDH1, GNA11, MLH1, SMO, CDKN2A, GNAS, MPL, SRC, CSF1R, GNAQ, NOTCH1, STK11, CTNNB1, HNF1A, NPM1, TP53, EGFR, HRAS, NRAS, VHL, ERBB2, IDH1, PDGFRA, ERBB4, JAK2* and *PIK3CA*. Multiplex PCR was also performed using the Ion AmpliSeq Colon and Lung Cancer Research Panel v2. This panel consists of a single pool of 92 pairs of primers and covers some of the genes of the Cancer Hotspot panel as well as some additional genes including *DDR2, MAPK1* and *FBX7*. Library preparation was performed using 10 ng of DNA according to the manufacturer's recommendations. The obtained DNA fragments were digested with FuPa followed by ligation of barcoded sequencing adaptors (Ion Xpress barcode adapters 1–16, Life Technologies) and purified using AMPure XP magnetic beads (Beckman Coulter, Nyon) according to the manufacturer's instructions.

Libraries were diluted to 1.2 ng/mL. 4 libraries of the Cancer Hotspot panel or 8 libraries of the Colon and Lung Cancer Research were pooled and subjected to emulsion PCR using the Ion OneTouch 200 bp kit (Life Technologies). After enrichment (Ion OneTouch ES), the library was sequenced using the Ion Torrent 200 bp kit (Life Technologies). Sequence analysis was performed on an Ion 316 chip v2 using an Ion Torrent PGM (Life Technologies). The average number of mapped reads was 480, 000 (range 323, 000–672, 000), the percentage of reads that were aligned over a target region was 96% (94–97%), the average mean depth was 2, 000 (1, 000–3, 200) and the average uniformity was 98% (95–100%). The average target base coverage at 100x was 99.4% (98–100%).

Raw data analysis was performed using the Ion Torrent Suite software 4.2 relative to the human reference sequence (hg19). For variant calling (v4.2.1.0) somatic low stringency configuration was used. Detection of single nucleotide polymorphisms or indel polymorphisms was performed using the Torrent Reporter 4.2 and the AmpliSeq CHPv2 single sample workflow. Alternatively, sequence alignment and variant detection was performed using the CLC Cancer Research Workbench version 1.5.2. Variant allele frequency threshold was set to 5%. Identified mutations were visually inspected using the IGV software.

### Sanger sequencing

Mutations identified by semiconductor sequencing were confirmed by Sanger Sequencing. To this end, DNA was extracted from laser capture microdissected material and amplified by PCR in a total volume of 30 μl containing 0.5 μM of the same primer pair as was used for library preparation for NGS and 1 Unit Hot Start Taq Polymerase (Qiagen) with an initial hold stage at 95°C for 10 minutes followed by 40 cycles of 94°C, 60°C and 72°C for 1 minute each. The sequence of the primers is available at the homepage from Life Technologies. PCR products were purified using the ExoSap-IT cleanup kit (Affymetrix) and subjected to sequencing using the Big Dye Terminator v1. 1 cycle sequencing kit (Applied Biosystems). Sequencing products were purified using the Big Dye x-Terminator purification kit (Applied Biosystems) and analysed using the Genetic Analyzer 3500 (Applied Biosystems). Sequence analysis was performed using the Sequencing Analysis Software 5.4 (Applied Biosystems).

### Real-time PCR

Real-time PCR was performed as described [39]. Quantitative PCR was carried out using TaqMan assay (Applied Biosystems) and the One-step PCR system (Applied Biosystems). The mean C_T_ was determined from triplicate experiments. miRNA levels were normalized to the level obtained for RNU48. Changes in expression were calculated using the ΔΔC_T_ method.

### Statistics

Statistical analyzes were performed using the GraphPAD prism software. Statistical differences were calculated using unpaired two-tailed Student's *t* test. A probability of *p* ≤ 0.05 was considered statistically significant.

## SUPPLEMENTARY FIGURE AND TABLES


