# Supplementary material for: Molecular profiling of lung adenosquamous carcinoma: hybrid or genuine type?
Source: Oncotarget. 2015 Jun 3;6(27):23905–16. doi: 10.18632/oncotarget.4163 (PMC4695160; doi:10.18632/oncotarget.4163)
Supplement: Supplementary file 1 [file oncotarget-06-23905-s001.pdf]

SUPPLEMENTARY FIGURE AND TABLES

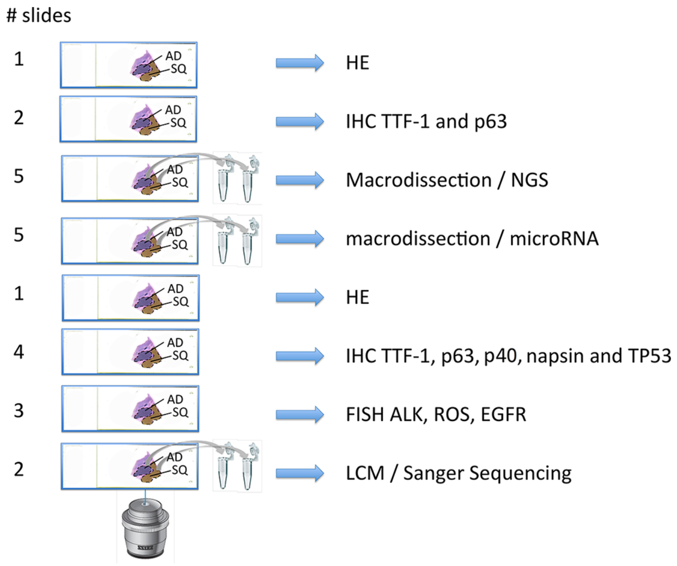

Supplementary Figure 1: Serial tissue sections of ADSQ.

Supplementary Table 1: Patient characteristics

		N = 16	%
Median age			
	years	75.7	
	range	57–84	
Gender			
	Male	12	75
	Female	4	25
TNM			
	pT1 pN0	2	15
	pT1 pN1	1	8
	pT2 pN0	4	30
	pT2 pN2	3	23
	pT3 pN0	1	8
	pT3 pN1	1	8
	pT4 pN0	1	8
Differentiation			
	well	1	7
	moderately	11	73
	poorly	3	20

Supplementary Table 2: Mutations identified by NGS

Tumor #	Gene	coverage AD/SQ	allele freq AD/SQ (%)	fold diff[1]	cDNA/gDNA	Protein	specificity
1	DDR2	1605/1297	62/31	2	c.1323G > A	p.M441I[2]	-
1	EGFR	2210/2459	43/52	1	c.2234_2245del AGGAATTAAGAG	p.E746_ E749del[2,3]	ADSQ
2	EGFR	3979/2359	22/67	3	c.2573T > G	p.L858R[2,3]	ADSQ
3	APC	1209/972	23/16	1	c.4106C > T	p.P1369V[2,3]	ADSQ
3	TP53	2312/1517	26/27	1	c.715A > G	p.N239D[2,3]	ADSQ
4	PIK3CA	2856/4014	8/1.6	5	c.1633G > A	p.E545R[3]	AD
4	TP53	1824/2494	26/0	> 10	c.464C > T	p.T155I[2,3]	AD
4	TP53	3694/4481	5/62	> 10	c.377A > G	p.Y126C[2,3]	SQ
5	STK11	1499/1083	34/8	4	chr19:1,220,371G > C	SV E4[2,3]	ADSQ
5	CDKN2A	564/386	49/0	> 10	c.329G > A	p.W110*[2,3]	AD
6	EGFR	866/2055	52/18	3	c.2573T > G	p.L858R[2,3]	ADSQ
7	APC	1281/2035	55/49	1	c.3949G > C	p.E1317Q[3]	ADSQ
7	JAK3	862/863	38/34	1	c.2164G > A	p.V722I[2,3]	ADSQ
8	TP53	4421/3587	66/59	1	c.747G > T	p.R249S[2,3]	ADSQ
8	CDKN2A	220/660	39/31	1	c.251A > G	p.D84G[2,3]	ADSQ
9	TP53	1896/1744	47/46	1	chr17:7578291A > T	SV E6[2,3]	ADSQ
10	PIK3CA	2021/2345	3/30	10	c.1633G > A	p.E545K[2,3]	SQ
12	PIK3CA	2636/2966	86/75	1	c.1258T > C	p.C420R[2,3]	ADSQ
12	RB1	1018/1824	58/29	2	c.1713_1714insTT	p.L572fs*39[2,3]	ADSQ
12	EGFR	1686/1919	34/27	1	c.2573T > G	p.L858R[2,3]	ADSQ
12	KIT	2687/3006	61/57	1	c.1621A > C	p.M541L[2]	-
13	TP53	2732/2583	11/44	4	c.569C > T	p.P190L[2,3]	SQ
14	KIT	175/400	44/63	1	c.1621A > C	p.M541L[2,3]	ADSQ
15	KIT	400/3711	45.8/81.1	2	c.1621A > C	p.M541L[2,3]	ADSQ
16	EGFR	459/397	87.3/86.4	1	c.2573T > G	p.L858R[2,3]	ADSQ
16	RB1	192/100	32.3/0	> 10	c.603_604insG	p.G203fs*[2,3]8	AD
16	PTEN	0/290	0/19	> 10	c.511C > T	p.Q171*[2,3]	SQ
16	TP53	400/400	67.5/58.5	1	c.814G > A	p.V272M[2,3]	ADSQ

[1]fold difference of allele frequency of AD and SQ components
[2]confirmed by Sanger sequencing
[3]somatic mutation
